# *Gynostemma pentaphyllum* Attenuates the Progression of Nonalcoholic Fatty Liver Disease in Mice: A Biomedical Investigation Integrated with In Silico Assay

**DOI:** 10.1155/2018/8384631

**Published:** 2018-03-21

**Authors:** Ming Hong, Zhe Cai, Lei Song, Yongqiang Liu, Qi Wang, Xiangfei Feng

**Affiliations:** ^1^Institute of Clinical Pharmacology, Guangzhou University of Chinese Medicine, 12 Jichang Road, Guangzhou 510405, China; ^2^Department of Orthopaedics and Traumatology, Li Ka Shing Faculty of Medicine, The University of Hong Kong, 21 Sassoon Road, Pokfulam, Hong Kong; ^3^Department of Cardiology, Xinhua Hospital, School of Medicine, Shanghai Jiaotong University, 665 Kongjiang Rd., Shanghai, China

## Abstract

Nonalcoholic fatty liver disease (NAFLD) is the most common type of liver disease in developed countries. Oxidative stress plays a critical role in the progression of NAFLD. Modern pharmacological study and clinical trials have demonstrated the remarkable antioxidant activity of* Gynostemma pentaphyllum* (GP) in chronic liver disease. One aim of this study was to explore the potential protective effects and mechanisms of action of GP extract on NAFLD. The* in vivo* results showed that GP extract could alleviate fatty degeneration and haptic fibrosis in NAFLD mice. For exploring the hepatoprotective mechanisms of GP, we used network pharmacology to predict the potential active components of GP and their intracellular targets in NAFLD. Based on the network pharmacology results, we further utilized biomedical assays to validate this in silico prediction. The results showed that Gypenoside XL could upregulate the protein level of PPAR*α* in NAFLD; the transcription level of several PPAR*α* downstream target genes such as acyl-CoA oxidase (ACO) and carnitine palmitoyltransferase-1 (CPT-1) also increased after Gypenoside XL treatment. The overexpression of ACO and CPT-1 may involve the hepatoprotective effects of GP and Gypenoside XL on NAFLD by regulating mitochondrial fatty acid *β*-oxidation.

## 1. Introduction

Nonalcoholic fatty liver disease (NAFLD) is a common chronic liver disorder which is related to insulin resistance and metabolic syndrome, such as obesity, diabetes, and hyperlipidemia [1, 2]. Oxidative stress is one of the key mechanisms responsible for disease progression in NAFLD [3]. Recent studies have shown that fatty acids could induce oxidative stress through regulating the expression or activity of key nuclear receptors such as PPAR*α*, *β*, *γ*, liver X receptor (LXR*α*), retinoid X receptor (RXR*α*), SREBP-1c, and others [4, 5]. Oxidative stress may produce peroxides and free radicals that further induce haptic cell damage and cell death in NAFLD patients [6–8].

*Gynostemma pentaphyllum* (Thunb.) Makino. (GP), a traditional Chinese medicine, has been reported to have hepatoprotective effect via numerous bioactivities, such as antifibrotic and antioxidative activity [9, 10]. GP is usually known as* Jiao Gu Lan* in China, the herb mainly contains polysaccharides, flavones, saponins, and trace elements. Recent research has demonstrated that GP extracts and its active components may protect alcoholic fatty liver disease in rats through modulating lipid metabolism and reducing oxidative stress. [11, 12]. Fuzheng Huayu tablet, a patented Chinese medicine formula from Shanghai Sundise TCM Co., Ltd., has successfully completed Phase II clinical trial in US FDA for treating liver fibrosis in patients with chronic hepatitis C [13, 14]. One of the main components in Fuzheng Huayu tablet is GP [15]. The mechanisms study showed that the protective effects of Fuzheng Huayu tablet may partly relate to the antioxidant activity of GP [15–17]. NAFLD are related to oxidative stress and fatty degeneration. Oxidative damage in the liver may cause lipid peroxidation and further induce hepatic cell death. Normally, liver can protect oxidative damage by its inner antioxidation systems such as the interacting network of antioxidant enzymes. Exogenous antioxidants such as some vitamins and natural supplements also can be effective in clearing reactive oxygen/nitrogen species (ROS/RNS) and inhibiting oxidative damage [18–21]. Previous researches have confirmed the protective effects of the GP on NAFLD on rat models [22, 23]. However, according to recent studies, mice models might more closely reflect the histopathology and pathophysiology characters of human NAFLD [24, 25]. In that case, we further explored the protective effect of GP on NAFLD mice and investigated the potential action mechanism in this study. Based on GP's significant antioxidant effects in alcoholic liver disease and haptic fibrosis, it may also exhibit potential hepatoprotective effects on NAFLD by preventing oxidative damage. For maximize the efficacy in the mechanisms study, network pharmacology will be used to analyze the biological network of GP and predict the underlying molecular mechanisms. Based on these in silico predictions, further experimental validation will be introduced to confirm the network pharmacological results and exploring the hepatoprotective mechanisms of GP.

## 2. Results

*HPLC-MS Analysis of Saponins and Flavonoids Fractions in GP*. A total of 10 saponins were separated and identified in GP (Table 1), the total content of saponins is 1301.2 *μ*g/mL, while Gypenoside XL (543.1 *μ*g/mL) and Gypenoside XXIII (339.7 *μ*g/mL) were the most abundant ingredients in total saponins of GP. Likewise, a total of 6 flavonoids were separated and identified (Table 1); the total content of flavonoids is 279.4 *μ*g/mL, while kaempferol (98.1 *μ*g/mL) and quercetin-rhamnose (49.3 *μ*g/mL) were the most abundant ingredients in total flavonoids of GP.

### 2.1. Biochemical Assays and Physiological Parameters Results of GP Treatment on Nonalcoholic Liver Disease

**Figure 1:**
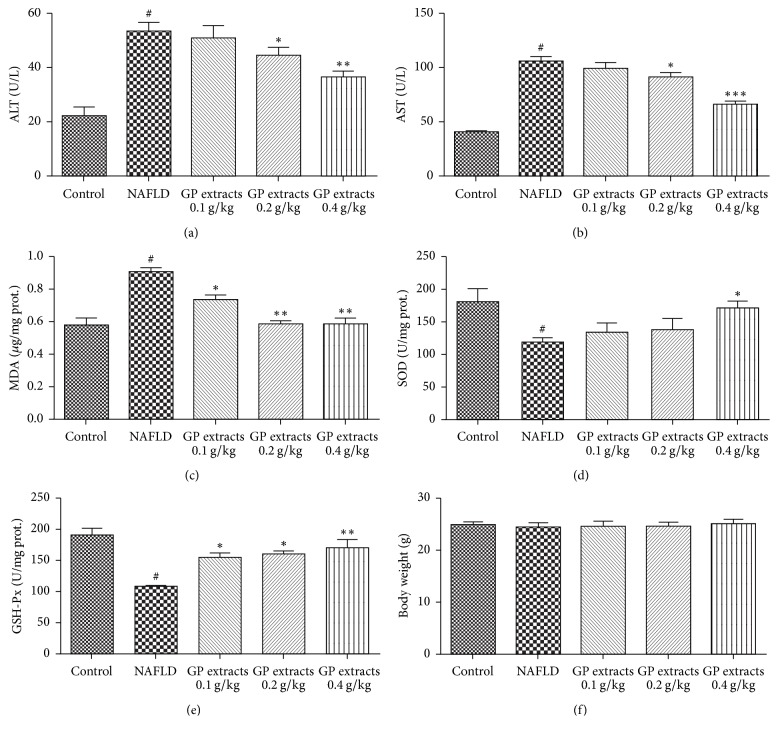
Biochemical assays and physiological parameters results of GP treatment on nonalcoholic liver disease. Treatments with GP extracts have no obvious effects on the body weight in NAFLD mice; treatment with GP extracts 0.2 g/kg/day and 0.4 g/kg/day decreased both serum ALT and AST level; GP could increase SOD, GSH-Px level in haptic tissues. In addition, MDA levels in NAFLD liver tissue were significantly increased compared with the normal group; GP extracts can alleviate the rise of MDA in mice liver (*p* < 0.01). ^#^Compared with normal group *p* < 0.05, ^*∗*^*p* < 0.05, ^*∗∗*^*p* < 0.01, compared with model group.

CDAA diet feeding slightly increased mice body weight in model group although there was no statistical significance. Treatments with GP extracts have no obvious effects on the body weight in NAFLD mice (Figure 1(f)). CDAA diet feeding combined with CCl4 injection significantly induced liver damage in NAFLD mice, as indicated by significant upregulated serum ALT and AST levels. Treatment with GP extracts 0.2 g/kg/day and 0.4 g/kg/day decreased both serum ALT and AST level in NAFLD mice (Figures 1(a) and 1(b)). The biochemical analysis results indicated that GP could increase GSH-Px and SOD level in haptic tissues (Figures 1(d) and 1(e)). In addition, MDA levels in NAFLD liver tissue were significantly increased compared with the control group; GP extracts can alleviate the rise of MDA in mice liver (*p* < 0.01) (Figure 1(c)). As the concentration of MDA represents the peroxidation extent of liver lipid, the rise of MDA concentration in hepatic cell indicated the distinct oxidative damage by NAFLD. GP extracts seemed be effective in alleviating the CDAA and CCl4 induced oxidative damage and further alleviate the hepatic injury.

### 2.2. Histological Results after GP Treatment on Nonalcoholic Fatty Liver Disease

**Figure 2:**
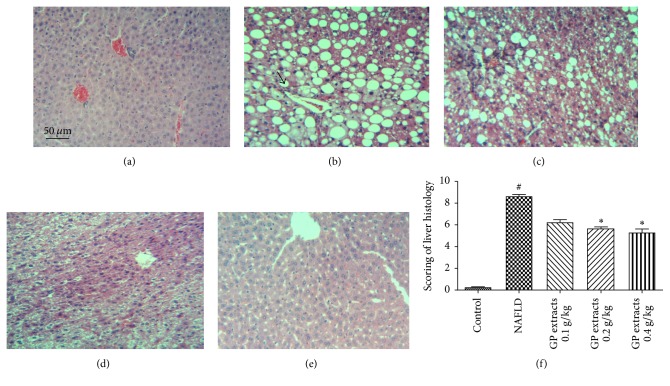
H&E staining results after GP extracts treatment in NAFLD (original magnification 100x). (a) The normal group had a clear structure of the hepatic lobule, and there were no visible lesions. (b) In the model group, typical pathological characteristics such as inflammatory infiltration (black arrow) and macrovacuolar degeneration can be observed. ((c)–(e)) Treatment with GP (0.093, 0.28, and 0.84 g/kg) for six weeks significantly attenuated the inflammation and macrovacuolar degeneration in the liver. (f) Scoring of liver histology of NAFLD mice with GP treatment (mean ± SD). ^#^*p* < 0.05, compared with normal group, and ^*∗*^*p* < 0.05, compared with model group.

**Figure 3:**
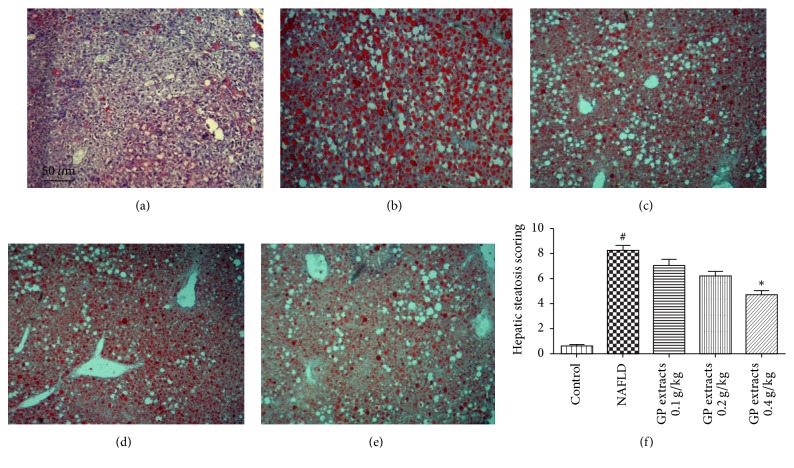
Oil Red O staining results after GP extracts treatment in NAFLD (original magnification 100x). (a) The normal group had no Oil Red staining. (b) In the model group, significant Oil Red stain which indicated steatosis can be observed. (e) Treatment with GP (0.4 g/kg/day) for six weeks significantly attenuated the steatosis in the liver. (f) Scoring of steatosis histology of NAFLD mice with GP treatment (mean ± SD). ^#^*p* < 0.05, compared with normal group, and ^*∗*^*p* < 0.05, compared with model group.

**Figure 4:**
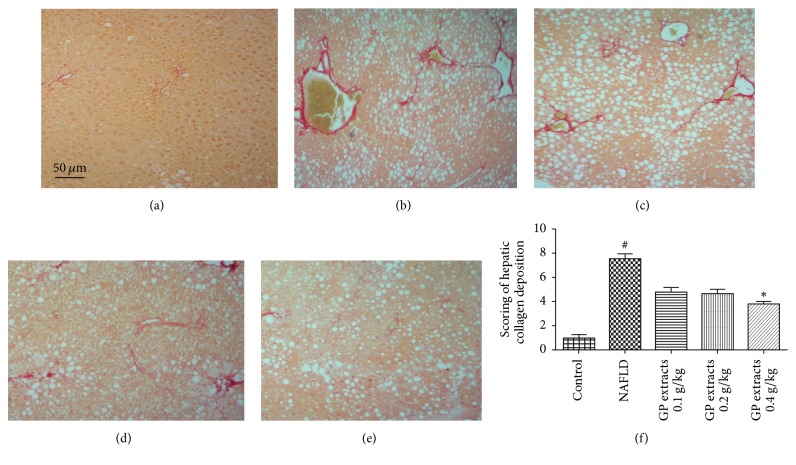
Picrosirius red staining results after GP extracts treatment in NAFLD (original magnification 100x). (a) The normal group had no visible Picrosirius red staining. (b) In the model group, significant Picrosirius red stain which indicated collagen deposition can be observed. (e) Treatment with GP (0.4 g/kg/day) for six weeks significantly attenuated the collagen deposition in the liver. (f) Scoring of liver fibrosis histology of NAFLD mice with GP treatment (mean ± SD). ^#^*p* < 0.05, compared with normal group, and ^*∗*^*p* < 0.05, compared with model group.

The histological results showed that CDAA chow diet feeding for 42 days combined with a low dose of carbon tetrachloride (CCl4) (0.4 *μ*L/kg b.w., twice/week) significantly induced fatty degeneration and hepatic fibrosis in mice liver. The H&E staining results demonstrated that 0.2 and 0.4 g/kg/day GP extracts treatment for 6 weeks significantly attenuated the inflammation and macrovacuolar degeneration in NAFLD (Figure 2). Oil Red O staining results showed that six weeks GP extracts treatment significantly attenuated the haptic steatosis in NAFLD mice (Figure 3). In addition, reduced scores of collagen deposition were found in GP treated groups, which indicated that GP treatment could reduce liver fibrosis in NAFLD mice (Figure 4).

### 2.3. In Silico-Based Network Construction and Analysis

**Figure 5:**
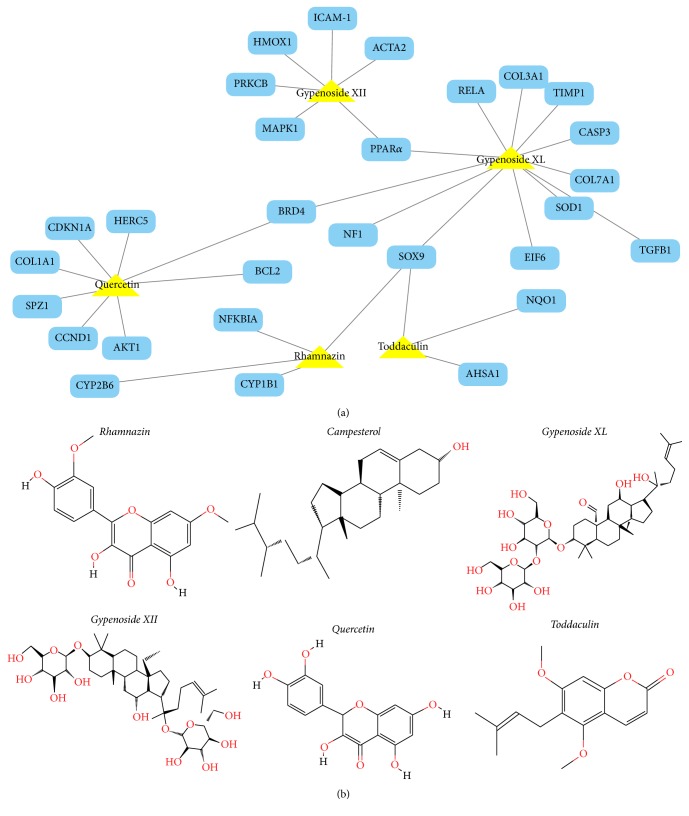
Compound-target networks and active compound structures in GP. The compound-target network related to NALFD was shown in (a). The yellow triangles are active compounds from GP and the blue rectangles represent potential NAFLD target genes; the gray line represents the compound-target interaction. (b) The corresponding chemical structures of the six potential anti-NAFLD ingredients from GP.

The potential active components of GP and their active mechanisms were predicted by in silico method. The drug-target network plotting showed the relationship between GP-derived chemicals and NAFLD related target genes or proteins (Figure 5). The results demonstrated that 6 potential active ingredients in GP exhibited favorable pharmacokinetics properties and may interact with different intracellular targets, which may relate with NAFLD. Among the 6 chemicals, Gypenoside XL, a water-soluble fraction from GP, showed the largest number (10) of connections with NAFLD related targets, followed by quercetin (8 connections) and Gypenoside XII (6 connections). For the 29 NAFLD related targets, the network results demonstrated that peroxisome proliferator-activated receptor alpha (PPAR*α*) had the largest number (3) of compound connections (campesterol, Gypenoside XII, and Gypenoside XL), followed by BRD4 (Gypenoside XL and quercetin) and SOX9 (Gypenoside XL and Rhamnazin). The other targets (26) showed interactions with just one chemical. Those high-degree nodes with multiple connections in the network, such as Gypenoside XL and PPAR*α*, BRD4, and SOX9, may develop multiple biological effects and play a more pivotal role in treating NAFLD. The data of 29 NAFLD related targets of GP can be obtained from Supplemental Table 1; all the information was collected from the STITCH, TTD, PharmGKB, and HIT databases.

### 2.4. Gypenoside XL Attenuated Haptic Injury in NAFLD Mice by Upregulating Protein Levels of PPAR*α* and Downstream Target Genes Related to Liver Lipid Metabolism

**Figure 6:**
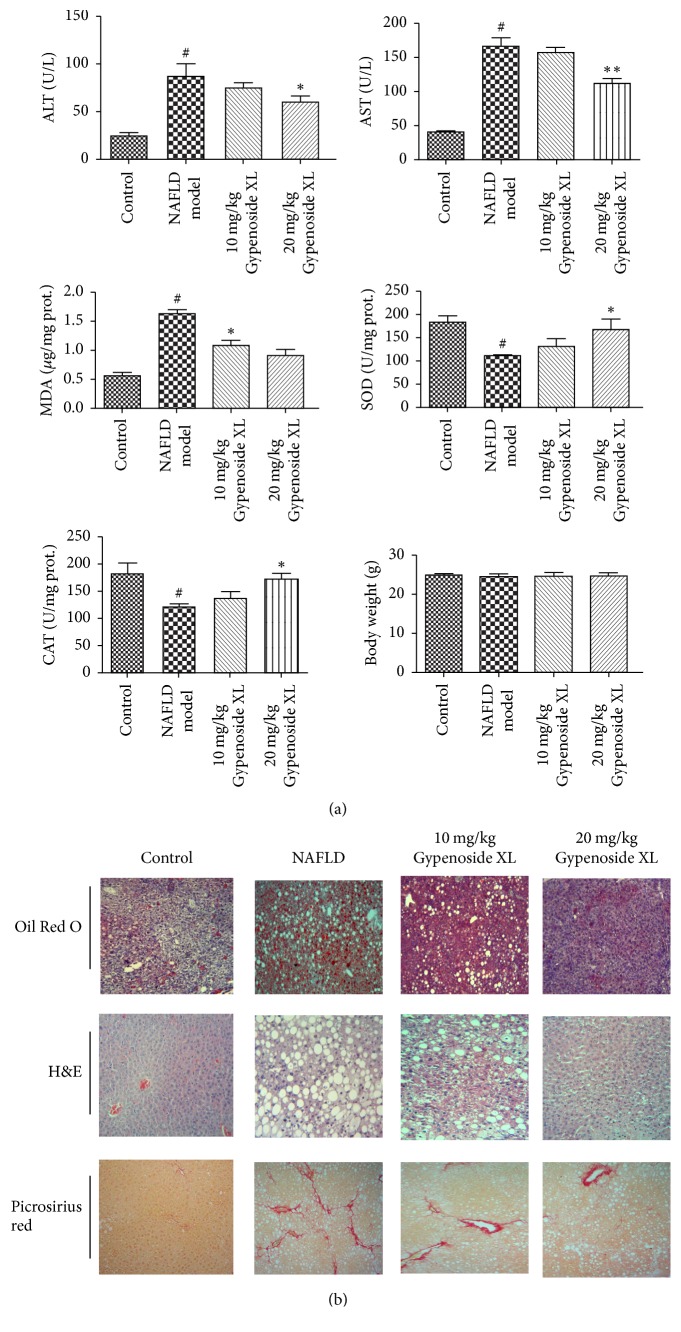
Gypenoside XL attenuated haptic injury in NAFLD mice. Treatments with GP extracts have no obvious effects on the body weight in NAFLD mice; treatment with Gypenoside XL 10, 20 mg/kg/day, decreased both serum ALT and AST level; Gypenoside XL could increase SOD, GSH-Px level in haptic tissues. MDA levels in NAFLD liver tissue were significantly decreased after Gypenoside XL treatment (*p* < 0.01) (a). The Oil Red O, H&E, and Picrosirius red staining results showed that Gypenoside XL 10 and 20 mg/kg/day could relieve haptic fat degeneration and fibrosis in NAFLD mice (b). ^#^Compared with normal group *p* < 0.05, ^*∗*^*p* < 0.05, ^*∗∗*^*p* < 0.01 compared with model group.

**Figure 7:**
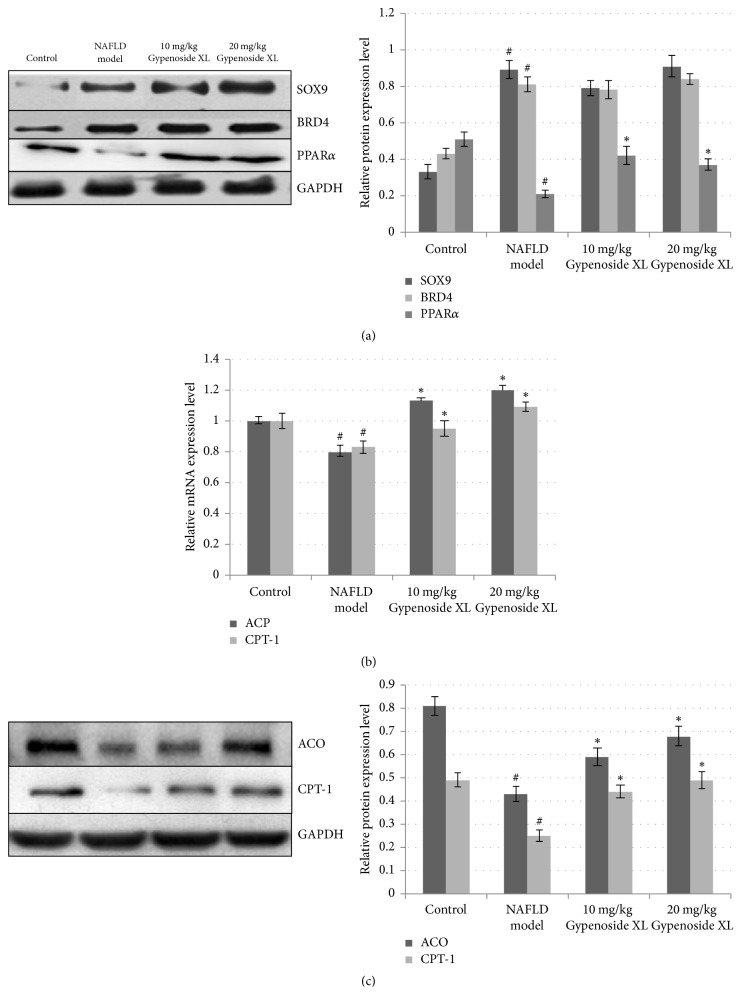
Gypenoside XL regulated PPAR*α* expression and downstream target genes related to liver lipid metabolism. In NAFLD mice, the haptic PPAR*α* protein level was significantly decreased after alcohol consumption. For SOX9 and BRD4 protein, the expression level was significantly increased. Gypenoside XL 10 and 20 mg/kg/day treatment led to a significant increase in PPAR*α* protein level compared with model group (*p* < 0.05). However, the expression of SOX9 and BRD4 protein was not affected by GP treatment (a). CDAA diet and CCl4 exposure can lead to downregulation of ACO and CPT-1 mRNA level as well as protein level in mice liver. Gypenoside XL 10 and 20 mg/kg/day treatment can upregulate mRNA and protein levels of ACO and CPT-1 in NAFLD mice ((b), (c)). ^#^*p* < 0.05, compared with normal group and ^*∗*^*p* < 0.05, compared with model group.

Gypenoside XL seemed to be effective in alleviating the CDAA diet and CCl4 induced oxidative damage and further alleviate the hepatic injury, which is confirmed by the biochemical assay results in serum and liver tissue (Figure 6(a)). The histological results showed that Gypenoside XL 10 and 20 mg/kg/day could relieve haptic fat degeneration and fibrosis in NAFLD mice (Figure 6(b)). Compared with the protective effects of GP extracts, Gypenoside XL 10 and 20 mg/kg/day have shown similar effects to GP in histological and biochemical assay results in NAFLD mice model. The reduction of PPAR*α* protein expression was observed in NAFLD mice (Figure 7(a)). For SOX9 and BRD4 protein, high-fat diet and CCl_4_ exposure can increase their protein expression. Gypenoside XL 10 and 20 mg/kg/day treatment led to a significant increase in PPAR*α* protein level compared with model group (*p* < 0.05). However, the expression of SOX9 and BRD4 was not affected by Gypenoside XL treatment. CDAA diet and CCl4 exposure can lead to downregulation of ACO and CPT-1 mRNA level as well as protein level in mice liver. Gypenoside XL 10 and 20 mg/kg/day treatment can upregulate mRNA and protein levels of ACO and CPT-1 (Figures 7(b) and 7(c)).

## 3. Discussion and Conclusion

Peroxisome proliferator-activated receptor alpha (PPAR*α*), a key node in our network pharmacology prediction, can modulate lipid peroxidation in fatty liver disease. PPAR*α* is a kind of nuclear receptor proteins; it is an important transcription factor for regulating the key enzymes in beta-oxidation pathway. Previous studies showed that PPAR*α* expression was downregulated in ALD and NAFLD in human liver [26]. Batatinha et al. showed that PPAR*α* can inhibit fatty liver disease by activating the periostin-dependent JNK signaling pathway and modulating fatty acid oxidation [27]. Activating PPAR*α* in liver can also prevent acetaminophen-induced liver damage by upregulating mitochondrial glutathione and downregulating fatty acyl-carnitines concentration in blood [28]. In addition, during chronic alcoholic liver injury, PPAR*α* also protect liver tissue by activating fatty acid beta-oxidation related pathway [26]. These researches demonstrated that PPAR*α* might be an important target for preventing NAFLD. Previous studies showed that several ingredients in GP such as Gypenosides may target PPAR*α* and regulate the expression of downstream genes in human endothelial cells and monocytic cells [29, 30]. In the present study, we have found that GP extracts could significantly relieve fatty degeneration and fibrosis in NAFLD mice liver. The decreased MDA level and increased antioxidative enzyme level indicated that GP can relive oxidative stress in NAFLD mice liver. Gypenoside XL, a potential active component in GP has shown similar protective effects to GP extracts in our NAFLD mice model. Further mechanism studies demonstrated that Gypenoside XL could up-regulate the protein expression level of PPAR*α* in NAFLD liver tissue. Although PPAR*α* has no direct effects to haptic lipid metabolism, it can up-regulate the expression of some key genes in mitochondrial and fatty acid *β*-oxidation [31]. To verify whether the raise of PPAR*α* expression can induce the transcription of *β*-oxidation related enzymes, the transcription level of several PPAR*α* downstream target genes such as acyl-CoA oxidase (ACO) and carnitine palmitoyltransferase-1 (CPT-1) was analyzed by RT-PCR and Western blot. Previous studies have shown that the expressions of ACO and CPT-1 were suppressed in NAFLD liver tissue, which is consistent with our results [32]. The upregulation of these two enzymes through increasing PPAR*α* expression may relate to the hepatoprotective effects of GP and Gypenoside XL on NAFLD.

In general, Gypenoside XL, a potential active component in GP may be a promising candidate phytomedicine against NAFLD. Network pharmacology study integrated with biomedical investigation may provide an efficient way to exploring the molecular mechanisms of herbal medicines. However, this in slico network pharmacology assay still needs further improvement with the rapid development of bioinformatics for providing a powerful tool for exploring the active mechanisms of herbal medicines and discovering novel bioactive ingredients.

## 4. Materials and Methods

### 4.1. Agents, Equipment, and Herb Extracts

Choline-deficiency amino acid-defined diet (CDAA) and control chow diet were obtained from Research Diets (New Brunswick, NJ, USA); carbon tetrachloride (CCl4) was purchased from Sigma-Aldrich (St. Louis, MO, USA); detection kits for alanine transaminase (ALT), aspartate transaminase (AST), malondialdehyde (MDA), superoxide dismutase (SOD), and glutathione peroxidase (GPx) are purchased from Jianchen Company (Nanjing, PRC); direct red 80 and picric acid were purchased from Sigma-Aldrich (St. Louis, MO, USA).* Gynostemma pentaphyllum* herb was collected from pharmacy dispensary of School of Chinese Medicine, Faculty of Medicine, the University of Hong Kong (HKU), and has been authenticated by Dr. Zhe Cai (Department of Orthopedics, HKU, Hong Kong, PRC). A voucher sample of the whole herb (SCM 127) was deposited at the School of Chinese Medicine, Faculty of Medicine, HKU, and the whole herb of* Gynostemma pentaphyllum* was cleaned and sliced into small pieces, after soaking for 3 hour and boiling for 1.5 hours under a high-heat fire. The filtered herbal residue was boiled again by the same method in above step. Then, all filtrate was collected, combined, and then concentrated at 60°C double distilled water to a final concentration of 0.5 g/mL. Gypenoside XL (PubChem CID: 94705-70-1) powder was purchased from Sigma-Aldrich Company in USA. Gypenoside XL was dissolved in Distilled Deionized Water and diluted in different concentration.

### 4.2. HPLC Analysis of Saponins and Flavonoids in GP

A method as described by Kao et al. was modified to prepare saponins and flavonoids from GP by open-column chromatography [33]. The various saponins in total saponin prepared from GP were separated with a Gemini HPLC column (Phenomenex, USA) and detected by Evaporative Light-Scattering Detector (Agilent, USA), followed by mass spectra determination and identification based on comparison of retention times and mass spectra, and fragmentation patterns of unknown peaks with authentic standards and those in previous publications. For quantitation, five concentrations of ginsenosides Rb3 and Rd (Sigma, USA) were prepared. Then, internal standard protopanaxatriol was added to each solution. The various saponins in total saponin were then calculated and quantified by utilizing a formula as described by Wu et al. [34]. The HPLC-MS analysis of flavonoids is similar to saponins; kaempferol (Sigma, USA) and rutin (Sigma, USA) were used as the flavonoid standards for analysis.

### 4.3. Animal Models and Treatment

For the establishment of NAFLD model, mice were fed with control chow or CDAA chow for 6 weeks. A low dose of carbon tetrachloride (CCl4) (0.4 *μ*L/kg b.w., twice/week) was used as promoter of hepatic fibrosis [35]. For the GP extracts treatment group, mice were orally given three doses of GP extracts (0.1, 0.2, and 0.4 g/kg/day) for 42 days. The dosage of GP extract was calculated from human dosage in clinic [36]. For Gypenoside XL extracts treatment group, mice were orally given three doses of Gypenoside XL (10, 20 mg/kg/day) for 42 days. Normal control group and model group of mice were treated with equal volume (0.2 mL) of saline water as placebo. 12 h later after last treatment, all mice were euthanized by cervical dislocation; serum and liver samples were immediately collected. All animal experiments were approved by the Committee on the Use of Live Animals in Teaching and Research of the University of Hong Kong. Approved number is 3658-15. The project starts on 11 July 2016. The animal license number in this study is (16-754) in DH/HA&P/7/1/2 Pt.46. This license was approved by Department of Health in Hong Kong on 20 April 2016.

### 4.4. Biochemical Assays

Blood samples were collected by cardiocentesis, the fresh blood was separated by centrifugation at 2000 rpm for 10 min, and then the serum was stored at −80°C until biochemical analysis. The serum levels of AST and ALT were analyzed with commercial kits according to the manufacturers' instructions (Jiancheng, Nanjing, PRC). Fresh liver tissue was sliced into small piece and homogenized in lysis buffer, centrifuged at 12,000 ×g for 5 min at 4°C, and the supernatants were collected and stored at −80°C until biochemical analysis. The activity of GSH-Px and SOD and the production of MDA were measured according to the manufacturers' instructions (Jiancheng, Nanjing, PRC).

### 4.5. Liver Histology

For hematoxylin and eosin staining, fresh mice liver tissue was sliced into 1.5 × 1.5 cm; then the tissues were fixed by formalin. The slides containing paraffin coated tissues were placed in a glass holder; then the slides were deparaffinized and rehydrated by different concentration of xylene and ethanol; then the slides were stained by hematoxylin for 5 minutes; after tab water washing and eosin staining for 30 seconds, the slides were dehydrated by different concentration of xylene and ethanol. All slides were covered by xylene based permount and stored in cool and dry environment. The histological results were analyzed by microscopy. The liver damage score was analyzed by two individual examiners with the following criteria: 0, no visible damage; 1–3, mild damage; 4–6, intermediate damage; 7–9, severe damage; and 10, destruction of hepatic structure. For assessing liver fibrosis, we have conducted Picrosirius red staining in hepatic tissue biopsy. The fresh liver tissue was fixed in formaldehyde solution for 48 hours. After paraffin embedding, the paraffin sections were dewaxed and hydrated by ethanol. Then, nucleus was stained with hematoxylin for 5 minutes, and then we used running tap water to wash the slides for 5 minutes. We stained the slides in Picrosirius red for 60 minutes and washed the slides in acidified water. Then, we shook the slides and dehydrated them in ethanol. Finally, the biopsy was mounted in a resinous medium and analyzed by microscopy. Hepatic fibrosis scoring on the stained sections was made by two individual examiners with the following criteria: 0, no signs of observed fibrosis; 1–3, no extension of portal area fibrosis; 4–6, fibrosis occurring in the portal area with an intact lobule structure; 7–9, fibrosis associated with a broken lobule structure, but no signs of cirrhosis; and 10, fibrosis and the formation of cirrhosis. The liver steatosis scores were evaluated by Oil Red O staining. Briefly, cryosectioned livers were washed with running tap water for 10 minutes, rinsed with 60% isopropanol, and stained with Oil Red O (Sigma, Cat# O0625-25G) mixed with 60% isopropanol for 15 minutes. The sections were then rinsed with 60% isopropanol, and nuclei were stained with Mayer's Hematoxylin, rinsed in tap water, and coverslipped with aqueous mounting medium. The liver steatosis score was analyzed by two individual examiners with the following criteria: 0, no visible steatosis; 1–3, mild steatosis; 4–6, intermediate steatosis; and 7–10, severe steatosis.

### 4.6. Molecular Database Construction

The chemical ingredients of GP were collected from following phytochemical databases: TCM Database@Taiwan (website: http://tcm.cmu.edu.tw/) and Traditional Chinese Medicine Systems Pharmacology Database (TCMSP) (website: http://lsp.nwu.edu.cn/tcmsp.php). The inconsistent data in these two databases were further verified and confirmed by related literatures review.

### 4.7. ADME Evaluation

A computer-based integrative model, ADME (absorption, distribution, metabolism, and excretion), was applied in this step to screen the chemicals with eligible pharmacokinetics properties in GP. PreOB (prediction of oral bioavailability) and PreCaco-2 (prediction of Caco-2 permeability) were major pharmacokinetics properties indicator in our ADME screening model. Oral bioavailability (OB) refers to the efficiency of the drug delivery to the blood circulation, which is an important property for oral medications. For calculating the OB value of GP-derived single compounds, OBioavail 1.1, a robust in silico model designed by Northwest A&F University, was applied in this step. This model was designed based on more than 800 structurally diverse drugs and drug-like agents. Support vector machine, multiple linear regression, and partial least square methods were used for building this model; the detailed algorithms of this model can be found in previous studies by Wang et al. [37]. Beside OB value, another important pharmacokinetics property for oral medications is the movement activity across the intestinal epithelium, which will affect the extent and rate of drug absorption as well as influencing its bioavailability. Herein, a preCaco-2 model was applied for predicting the efficiency of chemicals absorption in intestinal epithelium. The Caco-2 permeability information of the compounds was investigated through TCMSP database; the detailed screening parameters and algorithms can be found in TCMSP software (website: http://lsp.nwu.edu.cn/tcmsp.php). At last, ingredients meet the case that Caco2 ≥ 0.4 cm/s and OB ≥ 33% were chosen as candidate compounds for function analysis in the next step.

### 4.8. Identification of NAFLD Related Proteins and Genes

The biological activity of each single compound in GP was searched and analyzed using the software of Herbal Ingredients' Targets (HIT) Database (website: http://crdd.osdd.net/raghava/npact/) and Search Tool for Interactions of Chemicals and Proteins (STITCH) database (website: http://stitch.embl.de/); the information of ingredients and intracellular targets interactions retrieved from these databases was integrated and saved for further study. Then, for providing a more focused view on compound-NAFLD target interaction, we eliminated the unrelated target protein associations by mapping these targets to the Therapeutic Target Database (TTD) (website: http://bidd.nus.edu.sg/group/cjttd/). The above databases provide detailed information of the known or explored chemical agents and the corresponding intracellular targets and related human disease and pathologic changes. The protein targets related to NAFLD were further analyzed and confirmed by PharmGKB (website: http://www.pharmgkb.org) through using the following search terms: oxidative stress, fatty liver, hepatic steatosis, nonalcoholic liver disease, liver fibrosis, oxidative damage, and lipid metabolism.

### 4.9. Network Construction and Analysis

**Figure 8:**
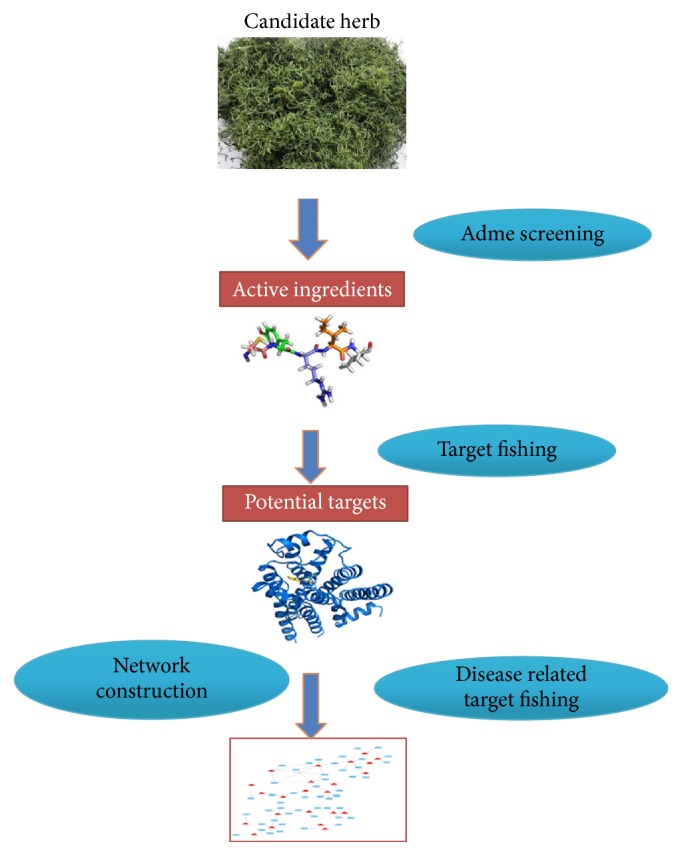
A flowchart of network pharmacology study of GP on NAFLD. The flowchart showed the procedures of network pharmacology study in our research. First step is building the herbal ingredients database, and then we used ADME screening to choose candidate chemicals and fishing their intracellular targets. After further NAFLD related target fishing, the data was processed by Cytoscape 3.4.0 and a network plotting was finally constructed for further analysis.

To provide a visible network between the GP-derived ingredients and NAFLD related protein targets, the most update Cytoscape 3.4.0 (website: http://www.cytoscape.org/) was used to construct the compound-target network plotting. In this visible network plot, a node stands for a chemical compound, gene or protein; the edge represents the compound-target interaction. The information of different nodes and their connections in the network were further analyzed by Network Analysis plugin to determine the key nodes for further experimental validate. The key nodes present the potential pivotal compounds or target proteins which may play important role in preventing NAFLD.  Figure 8 showed the complete procedure of network pharmacology study.

### 4.10. Western Blot

Liver tissue collected from normal control, NAFLD model, and Gypenoside XL treatment group mice was crushed by an Adjustable High-Speed Homogenate Machine with RIPA buffer on ice for 60 seconds and then centrifuged at 15,000 rpm at 4°C for 15 minutes. The protein concentration of the supernatant of liver homogenate was detected by BSA assay. Equal amounts of protein were resolved by SDS-Polyacrylamide gel electrophoresis and transferred onto Immun-Blot® PVDF Membrane. Then, use 5% BSA in TBST as blocking buffer to block the membrane at room temperature for 3.5 hours. The membrane was cut and each part was incubated with corresponding antibody (Santa Cruz, CA, USA) at 4°C overnight, then, after cleaning the membrane with TBST for 15 minutes for 3 times, the membrane was further incubated with corresponding secondary antibodies at room temperature for 3 hours. The immunoreactivities were tested by using Electrochemiluminescence Advanced kit and the results were analyzed with Bio-Rad chemi-luminescence imaging system. The protein expression levels of PPAR*α*, CPT-1, ACO, SOX9, and BRD4 were normalized with GAPDH and quantified.

### 4.11. Quantitative Real-Time PCR

Total RNA from liver tissue was extracted and purified using RNeasy Mini Kit following the manufacturer's instruction. QuantiTect Reverse Transcription Kit was used for reverse transcription of total RNA. The quantitative real-time PCR was conducted by using LightCycler 480 SYBR Green I master with 1 *μ*M primers (CPT-1 forward primer: gctgcgacgctcacagcgtg, CPT-1 reverse primer: acgactcgtacgcgaatcg; ACO forward primer ctcagctgaccacgttgctactcc ACO reverse primer: gaccatgtaaggccttcatg) in LightCycler 480 real-time PCR machine. The mRNA expression of *β*-actin (*β*-actin Forward primer: gccgtaccagataacctgtg, *β*-actin reverse primer: gcactaggcactcatctcg) was used as endogenous control. The relative expression of CPT-1, ACO mRNA for each sample was normalized with *β*-actin and the relative value was compared with normal control mice.

### 4.12. Statistical Analysis

All the data obtained are presented as the mean ± SD and analyzed by one-way analysis of variance (ANOVA) with Statistical Product and Service Solutions (SPSS) version 19 (IBM, Armonk, NY, USA). The significance of two groups was examined using a Student's *t*-test. Value of *p* < 0.05 was considered to be statistically significant.

## Figures and Tables

**Table 1:** Contents of saponins and flavonoids in GP (*μ*g/mL).

Fraction types	Chemical name	Content (*μ*g/mL)
Saponin	Gypenoside XL	543.1 ± 13.5
Saponin	Gypenoside XXIII	339.7 ± 8.8
Saponin	Gypenoside LXIb	62.3 ± 3.3
Saponin	Gypenoside LXIII	54.5 ± 2.7
Saponin	Gypenoside XXIIIb	46 ± 1.4
Saponin	Gypenoside-2	41 ± 0.6
Saponin	Gypenoside IV	40 ± 1.0
Saponin	Gypenoside VIII	37 ± 1.2
Saponin	Ginsenoside Rf	34 ± 1.5
Saponin	Gynoside A	25.2 ± 1.3
Flavonoid	Kaempferol	98.1 ± 11.4
Flavonoid	Quercetin-rhamnose	49.5 ± 7.9
Flavonoid	Caffeic acid	34.2 ± 5.3
Flavonoid	Quercetin-di(rhamnose)-hexoside	24.0 ± 4.9
Flavonoid	Rutin	21.7 ± 2.1
Flavonoid	Kaempferol-3-O-rutinoside	17.3 ± 1.5

## Supplementary Materials

Supplementary Materials Supplemental Table  1. The information of 29 potential targets in GP for NAFLD.
