# Supplementary material for: Gynostemma pentaphyllum Attenuates the Progression of Nonalcoholic Fatty Liver Disease in Mice: A Biomedical Investigation Integrated with In Silico Assay
Source: Evid Based Complement Alternat Med. 2018 Mar 21;2018:8384631. doi: 10.1155/2018/8384631 (PMC5884411; doi:10.1155/2018/8384631)
Supplement: Supplementary Materials — Supplemental Table 1. The information of 29 potential targets in GP for NAFLD. [file 8384631.f1.docx]

**Supplemental Table 1. The information of 29 potential targets in GP for NAFLD**

| **Target Gene** | **Target Protein** | **Organism** | **Roles in hepatoprotective effects** |
| --- | --- | --- | --- |
| SOX9 | Sex-determining region Y-box 9 | Homo sapiens | regulating of extracellular matrix deposition |
| CYP2B6 | cytochrome P450, family 2, subfamily B, polypeptide 6 | Homo sapiens | Oxidizes a variety of structurally unrelated compounds, including steroids, fatty acids |
| CYP1B1 | cytochrome P450, family 1, subfamily B, polypeptide 1 | Homo sapiens | Oxidizes a variety of structurally unrelated compounds, including steroids, fatty acids |
| BRD4 | bromodomain-containing protein 4 | Homo sapiens | Induce HSC activation into myofibroblasts |
| PPARα | peroxisome proliferator-activated receptor alpha | Homo sapiens | Key regulator of lipid metabolism |
| NFKBIA | nuclear factor of kappa light polypeptide gene enhancer in B-cells inhibitor, alpha | Homo sapiens | On cellular stimulation by immune and proinflammatory responses |
| AHSA1 | activator of heat shock 90kDa protein ATPase homolog 1 | Homo sapiens | Involve in Grb2-p38 MAPK signaling pathway in fibrosis |
| CYP1A2 | cytochrome P450, family 1, subfamily A, polypeptide 2 | Homo sapiens | Oxidizes a variety of structurally unrelated compounds, including steroids, fatty acids |
| NQO1 | NAD(P)H dehydrogenase, quinone 1 | Homo sapiens | Involve in alcohol detoxification pathways |
| HMOX1 | heme oxygenase (decycling) 1 | Homo sapiens | Alleviate liver inflammation and reduced oxidative stress |
| ICAM-1 | intercellular adhesion molecule 1 | Homo sapiens | Mediate adhesive interaction in fibrosis process |
| MAPK1 | mitogen-activated protein kinase 1 | Homo sapiens | Regulate cytoskeletal rearrangements in fibrosis process |
| PRKCB | protein kinase C, beta | Homo sapiens | Regulate oxidative stress-induced cell damage |
| ACTA2 | actin, alpha 2, smooth muscle, aorta | Homo sapiens | Involve in myofibroblast cell motility during wound healing in liver |
| SPZ1 | spermatogenic leucine zipper 1 | Homo sapiens | The transcriptional factors of liver fatty acid binding protein |
| COL1A1 | collagen, type I, alpha 1 | Homo sapiens | Transcriptional repressor of the collagen |
| BCL2 | B-cell CLL/lymphoma 2 | Homo sapiens | Regulate the response to mitochondrial damage and related oxidative damage |
| CCND1 | cyclin D1 | Homo sapiens | Functions as a mediator of β-catenin during hepatocarcinogenesis |
| HERC5 | HECT and RLD domain containing E3 ubiquitin protein ligase 5 | Homo sapiens | Acts as a positive regulator of innate antiviral response in liver cells |
| AKT1 | v-akt murine thymoma viral oncogene homolog 1 | Homo sapiens | Regulate lipid metabolism |
| CDKN1A | cyclin-dependent kinase inhibitor 1A | Homo sapiens | Regulate hepatic cell cycle in hepatocarcinogenesis |
| EIF6 | eukaryotic translation initiation factor 6 | Homo sapiens | Regulate hepatocarcinogenesis by mediating cellular response to DNA damage. |
| CASP3 | caspase 3 | Homo sapiens | Apoptosis inhibitory protein in hepatocarcinogenesis |
| COL7A1 | collagen, type VII, alpha 1 | Homo sapiens | Regulate fibrosis by impacts on extracellular matrix (ECM) proteins such as type IV collagen |
| COL3A1 | collagen, type III, alpha 1 | Homo sapiens | Regulate fibrosis by impacts on extracellular matrix (ECM) proteins such as type IV collagen |
| TGFB1 | transforming growth factor, beta 1 | Homo sapiens | Regulate liver cancer cells proliferation |
| TIMP1 | TIMP metallopeptidase inhibitor 1 | Homo sapiens | Tissue repair and induce interstitial fibrosis |
| SOD1 | superoxide dismutase 1 | Homo sapiens | Destroys radicals which are normally produced within the cells, such as oxidants |
| RELA | v-rel reticuloendotheliosis viral oncogene homolog A | Homo sapiens | Involve in hepatic inflammation |
